# Correlation between acanthosis nigricans and insulin resistance in obese children in Manado

**DOI:** 10.1186/1687-9856-2013-S1-P95

**Published:** 2013-10-03

**Authors:** Arief Gunadi, Vivekenanda Pateda, Adrian Umboh, Kristellina Sangirta Tirtamulia

**Affiliations:** 1 Department of Child Health, Faculty of Medicine, Sam Ratulangi University, DR R.D. Kandou General Hospital, Manado, Indonesia

**Keywords:** acanthosis nigricans, insulin resistance, diabetes mellitus type 2

## Background

Acanthosis Nigricans, which is a skin condition characterized by darkening and thickening of skin caused by papillomatosis and hyperkeratosis has been reported to be linked to insulin resistance and is thought to be a major factors in type 2 diabetes mellitus.

## Objective

To determine whether the presence of acanthosis nigricans in obese children is related with insulin resistance.

## Methods

We performed a cross sectional analytic observational study. One hundred twenty three obese children, ages 10 – 14 years with and without acanthosis nigricans got examined for insulin resistance using Homeostasis Model Assessment of Insulin Resistance Index (HOMA-IR). Diagnosis of acanthosis nigricans is confirmed by a dermatologist. This study took place in Wenang district, Manado, North Sulawesi from October 2009 until January 2010.

## Results

Acanthosis nigricans was found positive in 33 children (61.1%). We found insulin resistance in 84.4% of obese children with acanthosis nigricans. There was a positive correlation between acanthosis nigricans and obese children with insulin resistance (r=0.568, p<0.001).

## Conclusion

Children with acanthosis nigricans are more likely have insulin resistance. Therefore, we need to identify acanthosis nigricans in obese children for the possibility of diabetes mellitus type 2 so early intervention can be done.

